# Pro-inflammatory cytokine responses to *Naegleria fowleri* infection

**DOI:** 10.3389/fitd.2022.1082334

**Published:** 2022

**Authors:** Ching-wen Chen, E. Ashley Moseman

**Affiliations:** Department of Immunology, Duke University School of Medicine, Durham, NC, United States

**Keywords:** *Naegleria fowleri*, immune response to *Naegleria*, primary amoebic meningoencephalitis, CNS infection, olfactory infection, neuroinvasion, eukaryotic pathogen, immunopathology

## Abstract

*Naegleria fowleri*, or the “brain-eating amoeba,” is responsible for a rare, but lethal, infection known as primary amoebic meningoencephalitis (PAM). Confirmed PAM cases have seen both a rise in numbers, as well as expansion of geographic range over the past several decades. There is no effective therapy for PAM and the clinical prognosis remains grim with a mortality rate over 95%. The role of the immune response in disease prevention and disease severity remains unclear. In this review, we explore potential roles of inflammatory immune responses to *N. fowleri* in disease pathogenesis with a primary focus on pro-inflammatory cytokines IL-1, IL-6, and TNFα. We also discuss modulating proinflammatory cytokines as an additional immune therapy in PAM treatment.

## Introduction

*Naegleria fowleri*, also known as the “brain-eating amoeba,” is a free-living amoeba ubiquitously found in sources of warm fresh water around the world. *N. fowleri* belongs to the phylum Percolozoa, and is one of only four free-living amoebae genera that cause human disease (*Acanthamoeba* spp., *Balamuthia mandrillaris*, *Naegleria fowleri* and *Sappinia diploidea*) (1). Substantial genetic diversity exists within *N. fowleri* as eight genetic variations, or “genotypes,” have been identified around the world in geographically restricted ranges. Notably, only four genotypes have been reported in human infections (types 1, 2, 3, and 5). Because *N. fowleri* is prevalent in fresh water, human environmental exposure is very common, yet typically non-pathogenic. However, amoeba contact with the nasal cavity, particular the olfactory regions, can result in infection and a rare, yet fatal, disease called primary amoebic meningoencephalitis (PAM), first described by Fowler and Carter in 1965 (2). Naegleria infections are commonly linked to recreational water activity, but can occur in any situation in which contaminated water comes into contact with the host nasal cavity (including ritual ablution) (3, 4). Early PAM symptoms, including fever, headache, nausea, and vomiting, are shared by many illnesses, making it difficult to establish an early PAM diagnosis. The later symptoms of PAM include altered mental status, nuchal rigidity, seizures, and coma. The disease progresses rapidly and is typically lethal; worldwide, there are only 7 documented survivors out of 182 cases from 1961 to 2021 (5). In this review, we focus on the roles that pro-inflammatory cytokines play in the immune response to *N. fowleri* infection and propose targeting cytokines as a therapeutic approach in PAM treatment.

## Invasion of *Naegleria fowleri*

**FIGURE 1:**
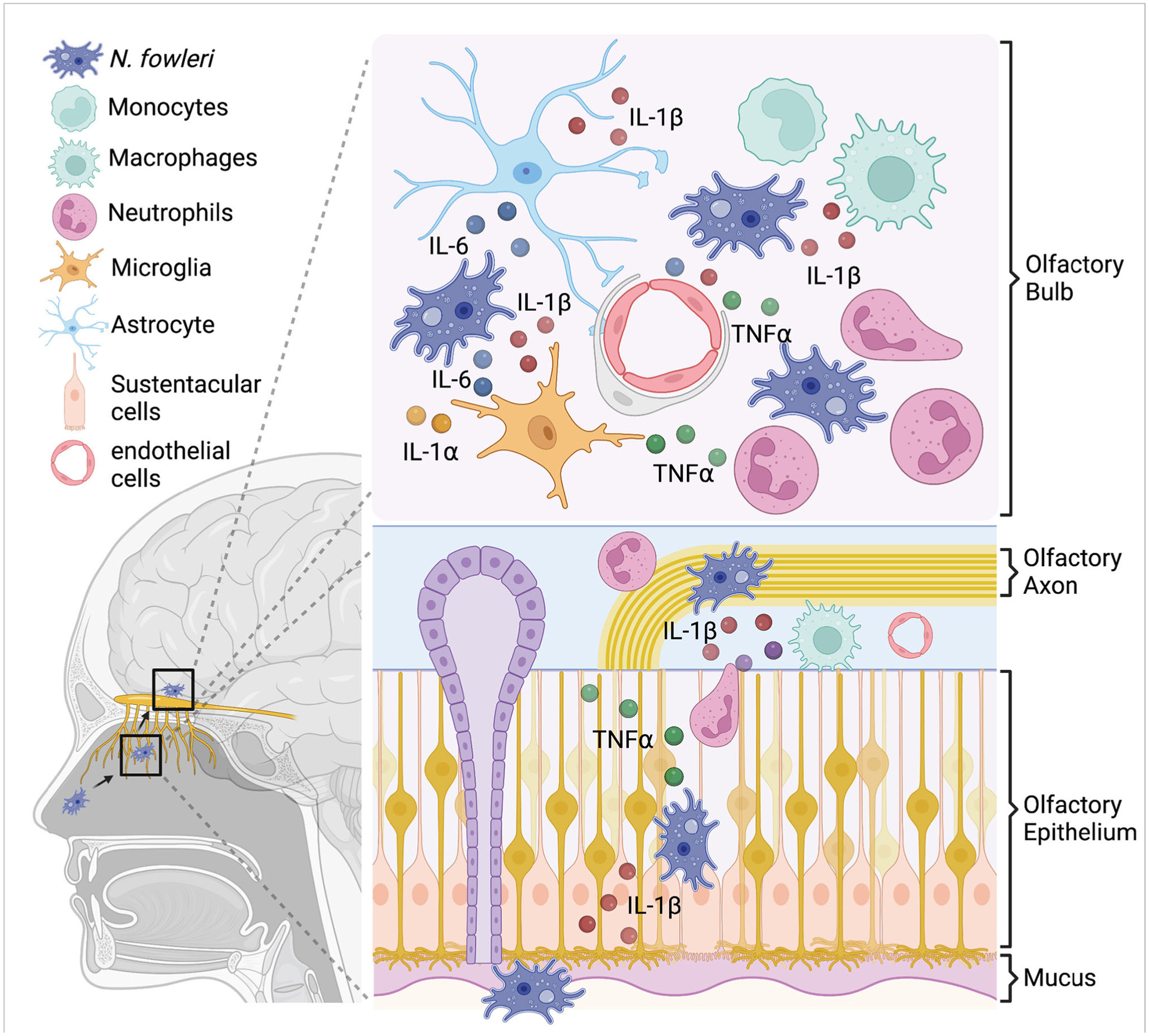
In vitro studies suggest a model for pro-inflammatory cytokine production during *N. fowleri* infection. *N. fowleri* attach to the olfactory epithelium and invade into the olfactory bulb through OSN axon tract. In the olfactory epithelium layer, resident macrophages and sustentacular cells may secrete pro-inflammatory cytokines in response to *N. fowleri*. In the olfactory bulb, microglia, astrocytes, endothelial cells, and macrophages may produce multiple pro- inflammatory cytokines after *N. fowleri* exposure. These cytokines likely play important anti- amoebic roles, but are also immunopathologic.

Once *N. fowleri* enters the nasal cavity, the amoeba must bypass several barriers within the turbinate structure and evade local immune surveillance to reach the olfactory bulb of the central nervous system (CNS). To start, the amoeba must reach the olfactory regions, located in the superior regions of the upper airway. At this point, the first line of defense is the mucus lying on the olfactory mucosa, secreted by Bowman’s glands (6). This mucus not only controls the ionic milieu of the olfactory sensory neurons (OSNs) but also provides a physical barrier to prevent direct pathogen attachment to the olfactory epithelium (7). Secretory IgA and IgM antibodies within the olfactory mucus could also impede pathogen movement in the olfactory tissues (8, 9). Anti-*N. fowleri* IgA antibodies have been reported in healthy human serum and saliva (10) at variable levels (11), however recent work from our group has indicated that serum antibody titers are poor prognostic indicators of olfactory protection (12). These data suggest antibody-based protection may, at best, be insufficient. Besides the pre-emptive defense potentially provided by antibodies within the nasal turbinates, an early cellular exudate in the mucus layer has been characterized in the mouse model of PAM. At 8h post-trophozoite inoculation, Rojas-Hernández and colleagues reported that immune cells and trophozoites were embedded in the mucus of the nasal cavity, suggesting an early cellular defensive mechanism to *N. fowleri* (13). However, the source of the immune cells and the mechanism of immune recognition of *N. fowleri* in the mucus layer remain poorly understood. Tight junction and adherens junctions lying between microvillar cells, supporting cells, and OSNs in the epithelium layers also establish a physical barrier to invasion within the nasal cavity (14). Nonetheless, *N. fowleri* trophozoites have been shown to destabilize the expression of tight junction proteins ZO-1, claudin-1, claudin-5, and occludin in MDCK cells and primary culture endothelial cells *in vitro* (15, 16). In the mouse model, *N. fowleri* trophozoites can invade the olfactory neuroepithelium without causing cell death or alarming the immune system at 24h post-infection (13). Notably, one recent *in vitro* study suggests that human mucoepithelial cells recognize *N. fowleri* trophozoites through TLR2/TLR4, and this further leads to pro-inflammatory cytokine production (17). Once the amoeba enters the lamina propria, it can readily gain access to OSN axon bundles. Olfactory sensory neuron axon bundles pass through the cribriform plate of the skull to enter the olfactory bulb within the CNS. The CNS has several effective barriers to prevent outside infiltration, however, these cribriform plate passages through the front of the skull, provide amoeba with a passageway into the brain that bypasses the blood brain barrier. It remains unclear how, or if, the amoeba selectively follows these axon bundles toward the brain. Some groups have suggested that neurotransmitters associated with olfactory neurons may act as a lure to attract *N. fowleri*. Indeed, there is evidence that a G-protein coupled receptor (GPCR) on *N. fowleri* surface has structural homology to the acetylcholine binding muscarinic acetylcholine receptor 1 (mAChR1) in humans (18). As *N. fowleri* invades deeper into the olfactory mucosa, entering the lamina propria and axon bundles (olfactory fascicles), it may encounter a unique population of extravascular neutrophils, that have been recently reported to uniquely surveil in the nasal epithelium and lamina propria under homeostatic condition (19, 20). Extravascular neutrophil encounter with amoeba could elicit a series of responses, including reactive oxygen or nitrogen species (ROS, NOS) production, and neutrophils extracellular trap (NET) formation within the olfactory tissues. Ultimately, in animal models, these effector functions can reduce the number of trophozoites invading the olfactory bulb of the CNS and slow fatal PAM development (21). But in animal models, amoeba invariably do reach the CNS and upon arrival glial cells have a minimal capacity to resist *N. fowleri* trophozoites. Microglia serve as the primary immune sentinel cell within the brain and these cells can respond to *N. fowleri* by activating NLRP3 inflammasome and MAPK signaling to secrete different pro-inflammatory cytokines (22). And while, published evidence suggests that microglia are capable of lysing and ingesting *N. fowleri* trophozoites, protease and extracellular vesicles secreted by *N. fowleri* can also lead to microglia cell death (23–25). As amoeba feed and divide, their increasing numbers result in damage that provokes an intense innate immune activation. Incomplete amoeba control initiates a feed forward loop of recruited blood-borne innate leukocytes and proinflammatory cytokine production that leads to a massively inflamed CNS environment with progressively increasing immune cell numbers (Figure 1). Innate immune cells are able to slow amoeba pathogenicity (21), and proinflammatory cytokines likely play important roles in their anti-amoebic pressure. Yet the excessive CNS immune response fails to control *N. fowleri* infection, and the collateral inflammatory damage leads to edema, hemorrhage, and elevated intracranial pressure that are critical factors in PAM pathogenicity. It’s unclear how individual proinflammatory cytokines are ultimately deleterious or beneficial to the PAM patients. Here we will further discuss the roles for proinflammatory cytokines, IL1 α/β, IL6 and TNFα in *N. fowleri* infection.

## Pro-inflammatory cytokines in *N. fowleri* infection

### IL-1α/β

The interleukin-1 (IL-1) cytokine family is a mainstay of innate immunity and inflammation. IL-1 signaling is a key feature of “inflammasome” activation and commonly viewed as a core pro-inflammatory cytokine. Indeed, IL-1 inhibitors have been clinically successful in treating autoimmune, as well as variety of inherited and acquired, inflammatory disorders. Two IL-1 family cytokines, IL-1α and IL-1β, are both reportedly produced by multiple cell types during *N. fowleri* infection (Table 1). While these IL-1 family members do not have direct effects on *N. fowleri* (31), they play critical roles in alerting or enhancing the immune reaction to *N. fowleri infection* [26].

Within the airway, nasal epithelial cells are one of first cell types to contact *N. fowleri* and *in vitro* studies suggest that they may respond with IL-1β secretion. A study of human mucoepidermal cell line found that IL-1β was secreted within 3h of co-culture with *N. fowleri* trophozoites. Interestingly, this study also found that trophozoites could induce mucin production from human epithelial cells (29). IL-1β secretion from monocytes or macrophages requires both priming (signal 1) and activation (signal 2) to produce activated cleaved IL-1β. Briefly, danger associated molecular pattern signals (DAMPs) activate the transcription factor NfκB (signal 1) leading to increased pro-IL-1β expression. Pro-IL-1β is an inactive precursor and requires cleavage by caspase-1 to form the active IL-1β molecule. Perturbation of ionic concentration (Ca^2+^ and K^+^ efflux) or further DAMP stimulation (32, 33) can lead to caspase-1 activation through the multi-protein inflammasome complex (inflammasome) (signal 2). In a non-contact co-culture system, macrophage-like cells (THP-1 cells) secrete cleaved IL-1β 3h post co-culture with *N. fowleri* trophozoites (22). Further analysis showed activation of caspase-1 and the ASC/NLRP3 inflammasome in THP-1 cells co-cultured with *N. fowleri* (22). Interestingly, another study found that *N. fowleri*-derived extracellular vesicles did not elicit IL-1α or IL-1β production from THP-1 cells (28). This finding implies that extracellular vesicles alone are minimally stimulatory, and damage caused by factors secreted by live amoeba is required to trigger IL-1 production. Overall, these *in vitro* data suggest that myeloid cells, especially, tissue-resident macrophages within the olfactory mucosa could respond to *N. fowleri* infection by secreting pro-inflammatory cytokines.

*In vitro* studies of multiple CNS cell types including microglia, astrocytes, and endothelial cells, have reported their secretion of IL-1α and IL-1β in response to *N. fowleri* infection. In one recent study, Lee et al. demonstrated that purified excretory and secretory *N. fowleri* proteins (NfESP) induced IL-1α and TNFα expression in BV-2 microglial cells (27). The same group showed that treatment of BV-2 microglial cells with a *N. fowleri* derived cathepsin B led to increased expression of IL-1α, IL-1β, IL-6, and TNF, and these effects are dependent on the NF-κB, AP-1 and MAPK signaling (26). Similarly, primary rat astrocytes also secrete IL-1β and IL-6 after being cultured with *N. fowleri* lysates and this secretion is dependent on ERK, JNK, and MAPKs activation (30). In addition to CNS glial cells, microvascular endothelial cells can express pro-inflammatory cytokines after contact with *N. fowleri* in an *in vitro* model; co-culture of *N. fowleri* with rat brain endothelial cells not only reduces endothelial tight junction protein expression but also increases expression of IL-6, IL-1β, and TNF (16). In the CNS, endothelial cells, astrocytes, and neurons, but not microglia, have been shown to homeostatically express IL-1R1 (34). During *N. fowleri* infection, abundant IL-1 release from different cell types may stimulate endothelial CXCL2 production that amplifies peripheral neutrophil recruitment and CNS immunopathology (34). Moreover, the elevated IL-1 may be responsible for “sickness behaviors” through endothelial IL-1R cell during *N. fowleri* infection (34). Unfortunately, current evidence is almost entirely based on data from *in vitro* systems and cell lines, and it remains unclear whether IL-1 signaling is essential to control *N. fowleri* infection *in vivo* and whether IL-1 signaling is ultimately beneficial or detrimental to the host.

### IL-6

IL-6 is a pleiotropic cytokine that is involved in the acute immune response and inflammation to infection. Experiments have demonstrated that IL-6 is produced by multiple cell types, including microglia, astrocytes, and endothelium cells after exposing to *N. fowleri in vitro* (Table 1). One critical role for IL-6 in infection is driving the host fever response through brain endothelium cells (35). The fever response is believed to benefit the host by generating a less hospitable environment for pathogens that thrive at or below body temperature. However, this is not the case for *N. fowleri*, which is a thermophilic organism that readily grows at temperatures above 40–42C. While IL-6 driven fever may not be an appropriate response to *N. fowleri*, IL-6R is highly expressed on neutrophils and monocytes (36), that are critical innate immune effectors that infiltrate the CNS during *N. fowleri* infection. Both monocytes and neutrophils can shed membrane-bound IL-6R from their cell surface to provide soluble IL-6R (sIL-6R) into the local environment (37, 38). Soluble IL-6R binds IL-6 to form a sIL-6R/IL-6 complex, that potently binds to GP-130 on endothelial cells, activating STAT3-dependent signaling, and inducing monocyte recruiting chemokine CCL2 expression (38, 39). Despite the known pro-inflammatory roles for IL-6 it is still unclear whether IL-6 can stimulate anti-amoebic function during *N. fowleri* infection or if the secondary effects contribute to tissue damage. More studies are needed to understand how IL-6 may impact *N. fowleri* pathogenesis.

## Tumor necrosis factor-alpha (TNFα)

TNFα has been frequently linked with anti-amoeba activities. In 1984, Ferrante and colleagues observed that conditioned medium from mononuclear leukocytes could activate human neutrophils to increase *N. fowleri* killing functions (40). TNFα has been shown to be the major player augmenting the neutrophil respiratory burst and lysosomal enzyme release in response to *N. fowleri* (41, 42). Myeloid cells express both TNFR1 and TNFR2, which bind soluble TNFα and membrane-bound TNFα, respectively. Several studies have reported TNFα “licensing” neutrophils to enhance or perform effector functions. TNFR1 signaling was shown to license mouse neutrophils and increase TLR-dependent cytokine production in a peritonitis model (43). Similarly, TNFα increased neutrophil elastase (ELANE) expression and induced NET formation *via* TNFR2 in human neutrophils (44), and NETs are reported to damage IgG opsonized *N. fowleri* trophozoites (45). In addition to myeloid cell activation, TNFα is known to potently increase endothelial cell adhesiveness (46), which in turn facilitates immune cell extravasation at inflamed sites. *In vitro* experiments have described TNFα production from microglia, endothelial cells, and epithelium cells upon *N. fowleri* stimulation. Notably, several *in vitro* studies have shown that TNFα release by microglia peaked 3h after *N. fowleri* exposure-earlier than observed IL-1β or IL-6 secretion (Table 1), suggesting that TNFα may contribute to the earliest immune activity in response to *N. fowleri* (24, 25, 27). TNFα clearly plays a key role in innate immune function, particularly through catalyzing subsequent inflammatory cascades, but more *in vivo* and mechanistic studies must be conducted to understand how TNFα signaling in different cell types and anatomical locations contributes to *N. fowleri* immunity.

## Targeting pro-inflammatory cytokines in PAM treatment

Initial symptoms of *N. fowleri* infection (headache and fever) belie disease seriousness. Even when meningitis is eventually suspected, delay in initiating treatments likely contributes to the fatality rate. Current clinical PAM treatment includes supportive care and broad-spectrum antifungals and antibiotics, including amphotericin B, fluconazole, rifampin, miltefosine, and azithromycin (47). External ventricular drain (EVD), hyperosmolar therapy, hyperventilation, and induced hypothermia have also been used to resolve cerebral edema and intracranial pressure in recent cases (5, 48). Nonetheless, the mortality of PAM remains over 95% and additional therapeutic approaches are desperately needed.

*N. fowleri* is not an obligate pathogen, and indeed infection is best described as opportunistic, but accidental (49). Mammalian immune systems are poorly adapted to handle the size and speed of *N. fowleri*, nevertheless, immune pressure does impede pathogen growth (21). Innate immune activation and inflammatory processes that arise within the CNS during amoeba infection are especially immunopathologic in the context of the confined CNS space. While the complex physiology of the CNS is impossible to mimic *in vitro*, a recent *in vitro* study indicated that leukocytes could enhance *N. fowleri*-induced cell death of human microvascular endothelial cells in co-culture experiments (50). This study reinforces the clinical use of anti-inflammatories for PAM patients and indeed all North American PAM survivors received dexamethasone, a corticosteroid with broad anti-inflammatory activities (5). The clinical utility of dexamethasone during PAM further suggests that the overwhelming brain inflammatory immune response is a critically pathologic component of disease. While the cerebrospinal fluid (CSF) from PAM patients has unfortunately not been analyzed for inflammatory mediators, many clinical studies have reported significant pro-inflammatory cytokine elevations during meningitis and encephalitis (51–55). These studies of CNS infections strongly suggest that PAM patient CSF contains high levels of pro-inflammatory cytokines. Targeted blockade of specific cytokines may therefore allow novel therapeutic approaches that alleviate detrimental inflammatory effects while preserving key immune-enhancing effects in PAM patients. Tocilizumab is a humanized monoclonal antibody targeting both membrane and soluble IL-6R, that is FDA approved by for severe cytokine release syndrome (CRS) (56) and has been issued severe COVID adults and pediatric patients (57). Tocilizumab delivery into the CSF space achieves significant *in vivo* concentrations in rhesus macaques, making it a candidate for controlling IL-6 signaling in PAM patients (58). IL-1α and β can be inhibited by recombinant human IL-1 receptor antagonist (Anakinra). Anakinra blocks IL-1α and IL-1β activities and is FDA approved for different inflammatory diseases, including rheumatoid arthritis, and cryopyrin-associated periodic syndromes (59, 60). Notably, the intravenous injection of Anakinra achieved effective concentrations in the CNS of subarachnoid hemorrhage patients, indicating blood-brain-barrier penetration (61).

TNFα blockade has been used clinically for over 20 years and five FDA approved drugs are currently available to block TNFα signaling (infliximab, etanercept, adalimumab, certolizumab pegol, and golimumab) for treatment of rheumatoid arthritis, psoriatic arthritis, and many other chronic inflammation diseases. During *N. fowleri* infection, excess TNFα within the CNS environments could make TNFα an appealing target for reducing inflammation. Indeed, perispinal TNFα blocking with etanercept improved stroke and traumatic brain injury (TBI) clinical outcomes in an observational study (62).

TNFα receptors (TNFR1 and TNFR2) have strikingly different downstream signaling that can drive seemingly contradictory CNS phenotypes. In neurons, TNFR1 activation is linked with neuroinflammation; while TNFR2 signaling is neuroprotective (63–65). The relative importance of TNFα signaling through either TNFR1 or TNFR2 during *N. fowleri* infection has not been fully elucidated. It will be necessary to dissect the *in vivo* TNFR1 and TNFR2 function during *N. fowleri* infection to determine if there may be clinical utility in TNFα blockade for PAM patients.

## Concluding remarks

While early identification of PAM remains a key clinical parameter, basic understanding of the immune response during *in vivo N. fowleri* infection is critical to improving current clinical outcomes. Here we have described what is known about pro-inflammatory cytokine secretion by different cell types in the context of *N. fowleri*. Billions of dollars have been spent to develop exquisitely targeted therapeutics against pro-inflammatory mediators that are now in widespread clinical use. These drugs offer opportunities to expand clinical treatment beyond the broad immunosuppression of corticosteroids, and selectively target detrimental components of the immunopathologic landscape during *N. fowleri* infection. However, additional *in vivo* mechanistic studies are needed to understand the specific beneficial and detrimental roles of the cytokine response in the CNS. *In vivo* studies of genetically deficient animals or with *in vivo* antibody blockade will be crucial to dissecting each cytokine’s role in the innate immune response and immunopathology during *N. fowleri* infection. Combining these *in vivo* studies with models of clinically relevant treatment approaches will support new therapeutic approaches to PAM.

## Figures and Tables

**TABLE 1:** Studies of pro-inflammatory cytokine response to *N. fowleri* stimulation.

Author	Experiment	Cell types	Stimulation	Findings
Oh, et al. (24)	In vitro co- culture	Rat primary microglia	co-cultured with *N. fowleri* trophozoites	IL-1β gradually increased after 12h, with no change after anti-Nfa1 antibody treatment. IL-6 secretion detected at 3h, increasing until 12h, with no change after anti-Nfa1 antibody treatment. TNFα production peaked at 3h and maintained similar levels after 12h, anti-Nfa1 antibody reduced TNFα secretion at 3h and 6h only.
Lê, et al. (26)	In vitro	BV-2 mouse microglial cells	Recombinant Cathepsin Bs of *N. fowleri* (rNfCBs)	IL-1α and IL-1β mRNA expression were detected at 6h after rNfCBs stimulation TNFα and IL-6 secretion were increased at 6h after rNfCBs stimulation Cytokine secretion was induced via NF-kB- and AP- 1-dependent MAPK signaling.
Lee, et al. (27)	In vitro	BV-2 mouse microglial Cells	Excretory and secretory proteins of *N. fowleri* (NfESP)	IL-1α and TNFα mRNA and protein expression increased at 3h post-NfESP stimulation. Cytokine production required NF-kB- and AP-1-dependent MAPK signaling.
Thái, et al. (23)	In vitro	BV-2 mouse microglial cells	recombinant fowlerstefin of *N. fowleri*	TNF, IL-1α, IL-1β, and IL-6 mRNA expression all increased 3h after recombinant fowlerstefin treatment. Cytokine production was downregulated by inhibition of NF-κB and AP-1.
Lee, et al. (25)	In vitro	Rat primary microglia	*N. fowleri* lysate	TNFα and IL-6 secretion was induced at 3h post-*N. fowleri* lysate stimulation. IL-1β expression was detected after 12h stimulation.
Kim, et al. (22)	In vitro co-culture	human macrophage	*N. fowleri* trophozoites	IL-1β secretion from THP-1 cells elevated at 3h after
		cells (THP-1 cells)	(non-contact system)	stimulation and ASC/NLRP3/Caspase-1 inflammasome was observed. In addition, NLRP3 and Caspase-1 inhibitors reduced IL-1β secretion from THP-1 cells after trophozoite co-culture.
Lertjuthaporn, et al. (28)	In vitro co- culture	human macrophage cells (THP-1 cells)	extracellular vesicles from *N. fowleri*	*N*. fowleri-derived extracellular vesicles drove THP-1 activation marker expression but not TNF, IL- 1α, IL-6, IL-10, and CXCL10expression.
Coronado- Velázquez, et al. (16)	In vitro co- culture	primary rat brain microvascular endothelial cells (RBMEC)	co-cultured with *N. fowleri* trophozoites	IL-1β, TNF, and IL-6 expression by RBMEC was detected after 6h co-culture with *N. fowleri*. Loss of tight junction proteins was found as early as 30 min of *N. fowleri* co-culture.
Cervantes- Sandoval, et al. (29)	In vitro co- culture	Human mucoepithelial NCI-H292 cells	co-cultured with *N. fowleri* at first 6h only	IL-1β mRNA levels were increased after 3h of co- culture with *N. fowleri*. In contrast, TNFα expression had no changes. ROS production and EGFR activation are required for IL- 1β expression. Mucin production was found after co-culture.
Martínez- Castillo, et al.(17)	In vitro cocultured	Human mucoepithelial NCI-H292 cells	co-cultured with *N. fowleri* trophozoites	IL-1β and TNFα mRNA expression were induced through TLR4 and TLR2 activation after 3h of *N. fowleri* exposure.
Kim, et al. (30)	In vitro	Primary rat astrocytes	*N. fowleri* lysate	IL-1β and IL-6 mRNA and protein expression were induced at 1h after *N. fowleri* lysate incubation. IL-1β and IL-6 secretion dependent on ERK, JNK and MAPKs activation.
